# Experimental study on creep properties prediction of reed bales based on SVR and MLP

**DOI:** 10.1186/s13007-021-00814-6

**Published:** 2021-10-30

**Authors:** Jixia Li, Lixin Zhang, Guangdi Huang, Huan Wang, Youzhong Jiang

**Affiliations:** 1 College of Mechanical and Electrical Engineering, Shihezi University, Shihezi, China; 2 Karamay Vocational and Technical College, Xinjiang, China

**Keywords:** Reed, Creep, Viscoelasticity, Machine learning, Prediction

## Abstract

**Background:**

Reed has high lignin content, wide distribution and low cost. It is an ideal raw material for replacing wood in the paper industry. Reeds are rich in resources, but the density of reeds is low, leading to high transportation and storage costs. This paper aims to study the compression process of reeds and the creep behaviour of compressed reeds, and provide theoretical guidance for the reed compressor management, bundling equipment and the stability of compressed reed bales.

**Results:**

We have established a multi-layer perceptron network prediction model for the creep characteristics of reeds, and the prediction rate R^2^ of this model is greater than 0.997. The constitutive equation, constitutive coefficient and creep quaternary model of the reed creep process were established by using the prediction model. The creep behaviour of the reed bale is positively correlated with the initial maximum compressive stress (*σ*_*0*_). During the creep of the reed, the elastic power and the viscous resistance restrict each other. The results show that the proportion of elastic strain in the initial stage is the largest, and gradually decreases to 99.19% over time. The viscoelastic strain increases rapidly with time, then slowly increases, and finally stabilizes to 0.69%, while the plastic strain accounts for the proportion of the total strain. The specific gravity of the reed increases linearly with the increase of creep time, and finally accounts for 0.39%, indicating that as time increases, the damage of the reed's own structure gradually increases.

**Conclusions:**

We studied the relationship between the strain and time of the reed and the strain and creep behaviour of the reed bag under different holding forces under constant force. It is proved that the multi-layer perceptron network is better than the support vector machine regression in predicting the characteristics of reed materials. The three stages of elasticity, viscoelasticity and plasticity in the process of reed creep are analysed in detail. This article opens up a new way for using machine learning methods to predict the mechanical properties of materials. The proposed prediction model provides new ideas for the characterization of material characteristics.

## Background

In recent years, over-cultivation of forests and pastures has led to enormous environmental problems [1, 2]. Due to their high lignin content, wide distribution, and low cost, reeds are an excellent candidate for a raw material to replace timber in the papermaking industry [3, 4]. Reed resources are abundant but have extremely low use efficiency, mainly because reed processing sites are relatively distant from reed fields, and reed bales prepared for transportation are low in density [5, 6]. These factors result in high transportation and storage costs. Therefore, it is necessary to study the reed compression process and the creep behaviour of compressed reeds to provide theoretical guidance on reed compression mechanisms, baling equipment and the stability of compressed reed bales [7, 8].

To date, numerous researchers have conducted extensive studies on the mechanical properties of viscoelastic materials. Reynolds et al. [9] examined the effects of compressive load and particle size on the compressibility of various varieties of smashed wheat straw. Krstic et al. [10] investigated the compression properties of treated corn stover, grassland rush and switchgrass. These researchers found that when the moisture content is 16–20%, the yield strength of these materials is relatively low, but the particles of these materials have a higher compaction density. Maraldi et al. [11] studied the effects of material, bale density, bale orientation, baling process and loading rate on the mechanical properties of bales. Kashaninejad M et al. [12] established a generalized Maxwell model and used this model to analyse the effects of lignocellulose content on the stress relaxation behaviour of 21 varieties of wheat straw. Nona and MD Shaw [13, 14] analysed the stress relaxation behaviour of straw under closed compression using a generalized Maxwell model. Maraldi et al. [11, 15] conducted creep and stress relaxation tests on straw bales moulded under compression. Based on the test results, these researchers established a mathematical model for straw bales. Additionally, these researchers noted that the creep properties of straw bales were directly proportional to the load. Through rheological tests on rice seedling stems, Scharenbroch et al. [16] concluded that the occurrence of a creep process and the plastic strain were positively correlated with the creep time and initial stress affecting the rice seedling stems. These studies demonstrate that the compression of straw bales is time-dependent [17, 18]. However, analytical studies on the post-compression creep properties of tall, thick and hard stalk crops (e.g., reeds) have yet to be reported.

The plant material compression process is complex, multivariate and unpredictable [19]. Traditional prediction methods are easy to implement [19, 20], but cannot accurately predict nonlinear systems. In addition, adjusting the parameters is troublesome and time-consuming [21, 22]. At the same time, the problem of model predictive control for nonlinear and time-varying uncertain systems has not been well resolved [23, 24]. As a commonly used prediction method, machine learning has good nonlinear characteristics [25, 26], convergence and a certain generalization ability. Therefore, machine learning technology has been widely used in the agricultural field in recent years [27, 28], such as the study of material characteristics [29, 30], the control of compression mechanisms [31, 32] and the inspection of work quality [33, 34].

In order to understand the dynamic mechanical properties of the reed bales more truly, a creep model of the reed blocks after compression is established, and the compression process of the reed bales is analyzed to provide theoretical guidance for the design of the reed baling mechanism. This paper first analyses the feasibility of using machine learning methods to predict the compression and creep deformation of reeds with highly nonlinear characteristics under the conditions of changing the compression time, strain value, stress and delay time of the material. Based on the experimental results again, a multi-layer perceptron (MLP) network and support vector machine (SVM) regression algorithm are used to establish a predictive model. Finally, the accuracy and stability of the model are studied by comparing the fitting performance of the training set of the model and the prediction performance of the new conditions. This article opens up a new way to predict the long-term mechanical properties of polymers through machine learning methods. In addition to various superposition principles, its biggest advantage compared to traditional models is that it can simulate the nonlinear characteristics of reed mechanics, it can also reduce the number of experimental conditions, shorten the experimental period, and provide ideas for the accelerated characterization of long-term mechanical properties of materials.

## Materials and methods

### Materials

**Fig. 1:**
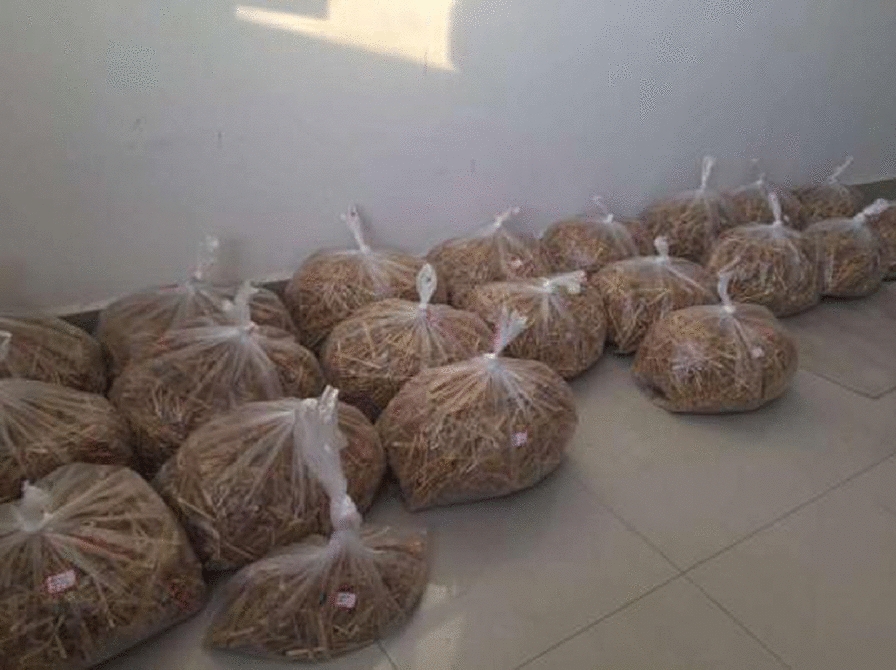
Experiment material

The reed samples used in this study were collected from Bosten Lake in the winter of 2018, as shown in Fig. 1. Table 1 summarizes the physical properties of the reed samples. The compression test results showed that the optimum compression with relatively low energy consumption was achieved with reeds that were 0.10 m in length and had a moisture content of 17%. On this basis, the long reed stalks harvested were cut into 0.10 ± 0.02 m short reed stalks. Before the tests, the reed samples were air-dried in a dry environment for more than a month. When determining the moisture content of the reeds, each reed sample was first dried in an electric blast drying oven. Subsequently, the mass (${m}_{1}$) of the reed was determined.

**Table 1:** Physical properties of the reed stalks

Whole-reed height (m)	Sample length (m)	Wall thickness ($\times {10}^{-4}$ m)	Cross-sectional area ($\times {10}^{-5}$ m^2^)	α (%)
3.50 ± 0.50	0.10 ± 0.02	9.50 ± 0.50	9.00 ± 0.10	17.00 ± 0.15

Spray evenly according to the required moisture content (α), with a mass of *m*_*2*_. Put the water into a sealed bag, classify and mark it, and let it stand for 48 h.

1 $$a = \frac{{m_{2} }}{{m_{1} + m_{2} }} \times 100{\text{\% }}$$

where α is the moisture content, ${m}_{1}$ is the mass of the reeds after drying, and ${m}_{2}$ is the mass of water.

### Test equipment

**Fig. 2:**
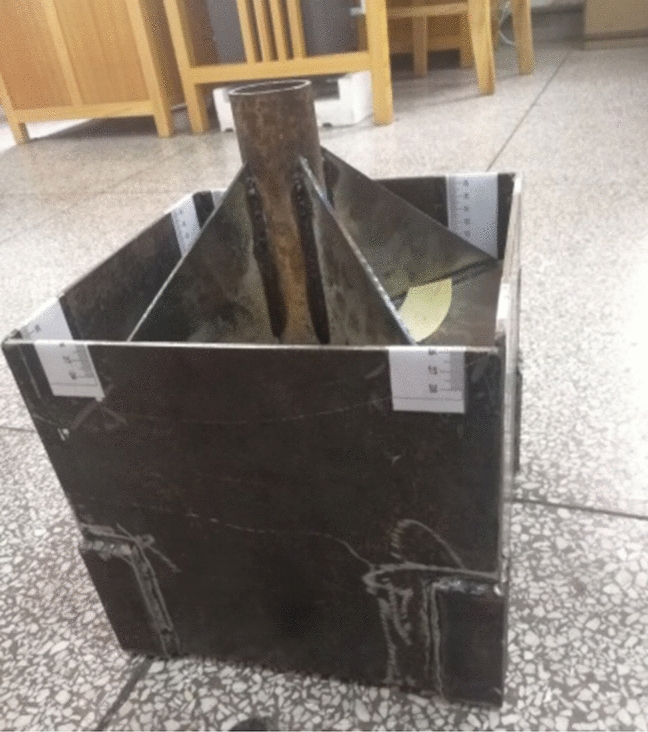
Compression apparatus

The creep properties of the reed samples were studied under closed compression conditions using a compression apparatus developed in-house (as shown in Fig. 2). The compression apparatus, made of 0.014-m-thick #45 steel sheets, consists of a piston rod, a compression cover plate, and a compression box. The compression box has inner cross-sectional dimensions of 0.20 × 0.20 m^2^. The piston rod is 0.15 m in length. Four ribbed plates were added onto the compression cover plate to prevent the plate from deforming under stresses. To facilitate the observations during the test, four rulers were adhered onto the inner walls of the compression box.

**Fig. 3:**
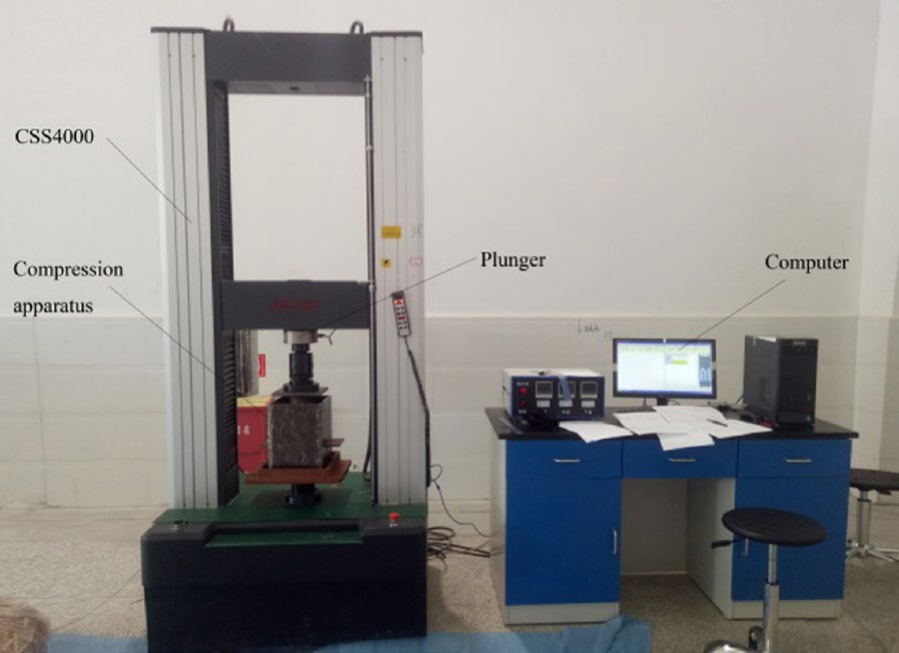
Compression test system

A UTM5305 computer-controlled electronic universal material testing machine (Fig. 3) was used in the tests. This machine has a maximum test force of 300 kN, a force measuring the precision of 1 N and a stress precision of 0.001 MPa. Additionally, an electric blast drying oven, an electronic balance, and a Vernier calliper were used in the tests. Moreover, according to the test requirements, an EDC120 digital control system, a GWB-200 high-precision displacement calibration instrument, and an extensometer were used in the tests. Auxiliary tools used in the tests included scissors, a Vernier calliper, a spray bottle, and a straightedge.

### Creep tests

A creep test examines the strain ($\varepsilon$)–time (*t*) relationship under a constant stress ($\sigma$). In essence, creep is a delayed material deformation process. The compression test results showed the following. For 0.05-, 0.10-, 0.15- and 0.20-m reed samples, the 0.10-m reed samples had the highest compaction density after compression under the same conditions (feed quantity, *α* and $\sigma$). The higher α was, the higher the compaction density was. However, high-α reeds could become mouldy, resulting in a decrease in the nutrient content of the reeds. Pre-test results showed that optimum compression was achieved for reeds with an *α* of 17%. In this study, the effects of $\sigma$ on the creep properties were analysed. Therefore, reeds with a length of 0.1 m and an *α* of 17% were selected. These reeds were randomly and evenly placed in test moulds. The reeds in each mould had a mass of approximately 1.150 ± 0.05 kg. The test location was marked. During the tests, the reeds were compressed vertically at a loading rate of 1.67 × 10^–3^ m/s. Once the set pressure was reached, a constant $\sigma$ was maintained for 800 s. Subsequently, the compression and creep tests were terminated. Table 2 summarizes the test conditions for each group of tests. At least three tests were conducted under each set of conditions. The final test results were averaged after eliminating relatively large errors. All the tests were conducted at a room temperature of 25.2 ± 2.2 °C and a relative humidity of 46 ± 4%.

**Table 2:** Creep test conditions

Test No	Test reed sample length (m)	α (%)	Loading rate (m/s)	Maximum compressive $\sigma$ (Pa)	Retention time (s)	Feed quantity (kg)	Bale orientation
1	0.10	17	1.67 $\times 10^{ - 3}$	3 $\times 10^{6}$	800	1.150	Flat
2	0.10	17	1.67 $\times 10^{ - 3}$	4 $\times 10^{6}$	800	1.150	Flat
3	0.10	17	1.67 $\times 10^{ - 3}$	5 $\times 10^{6}$	800	1.150	Flat
4	0.10	17	1.67 $\times 10^{ - 3}$	6 $\times 10^{6}$	800	1.150	Flat
5	0.10	17	1.67 $\times 10^{ - 3}$	7 $\times 10^{6}$	800	1.150	Flat

### Data analysis

#### Input andoutput variables of the model

The creep properties of a linear viscoelastic material are generally described using the Burgers four-element model, which consists of a Maxwell model and a Kelvin model connected in series. Equation (3) shows the constitutive equation for the Burgers four-element model.

2 $$\varepsilon \left( t \right) = \frac{{{\upsigma }_{0} }}{{E_{m} }} + \frac{{{\upsigma }_{0} }}{{E_{k} }} \times 1 - {\text{e}}^{{ - \frac{{E_{k} }}{{\eta_{k} }}{\text{t}}}} + \frac{{{\upsigma }_{0} }}{{\eta_{m} }} \times t$$

where $\varepsilon \left( t \right)$ is the $\varepsilon$ at the time *t* (%), $\sigma_{0}$ is the initially applied stress (Pa), ${\text{E}}_{{\text{m}}}$ is the instantaneous elastic modulus (Pa), ${\text{E}}_{{\text{k}}}$ is the delayed elastic modulus (Pa), ${\upeta }_{{\text{k}}}$ is the coefficient of delayed viscosity (Pa × s), and ${\upeta }_{{\text{m}}}$ is the coefficient of viscosity (Pa × s).

Equation (4) can be used to analyse the proportion of each type of $\varepsilon$ in the ${\text{v}}_{c}$ of reeds.

3 $$P_{i} t = \frac{{\varepsilon_{i} \left( t \right)}}{\varepsilon \left( t \right)} \times 100{\text{\% }} = \frac{{J_{i} \left( t \right)}}{{J_{e} \left( t \right) + J_{ve} \left( t \right) + J_{v} \left( t \right)}} \times 100{\text{\% }}$$

The factors affecting the compression performance of reed include compression time, stress, strain and delay time. In order to reflect the elastic strain, viscoelastic strain and viscous strain of reed compression, the compression time *t*, strain value $\varepsilon \left( t \right)$, stress $\sigma_{0}$ and delay time *T*_*K*_ are taken as the input $x = \left[ {t,\varepsilon \left( t \right),\sigma_{0} ,T_{K} } \right]^{T}$, and the elastic creep compliance *J*_*e*_, viscous creep compliance *J*_*ve*_ and plastic creep compliance *J*_*v*_ are taken as the output.

#### Data normalization

The range of input variables and strain results of the creep experiment showed that the input and output variables were not in the same order of magnitude. However, multiple machine learning methods, such as ANN, require that the weights and other parameters in the model are parallel in order of magnitude. If the difference of input variables is large, the input variables with a smaller order of magnitude will be covered by those with a larger order of magnitude during the error propagation. Furthermore, the effect of each input variable on the output cannot be rendered properly. Consequently, normalizing the input and output variables is crucial before modeling.

In this research, the z-score method, which is commonly used alongside machine learning, was considered to normalize the input and output variables, so that the mean value of each variable equals 0 while the variance equals 1. The method can be expressed as

4 $$\hat{x}^{\left( x \right)} = \frac{{x^{\left( x \right)} - \hat{x}}}{S}$$

where *x*^(*n*)^are the original samples, *n* = 1, 2,…, *N*, and *N* is the number of samples, x is the mean value of samples, *S* is the variance of samples, $\hat{x}^{\left( x \right)}$ are the normalized samples. After normalization, the input variables can be expressed as $\hat{x} = \left[ {\hat{t},\hat{\varepsilon }\left( t \right),\hat{\sigma }_{0} ,\hat{T}_{K} } \right]^{T}$, while the output variables change to $\hat{J} = \left[ {\hat{J}_{e} ,\hat{J}_{ve} ,\hat{J}_{v} } \right]$.

#### Multilayer perception network

Multilayer Perceptron Network (MLP) is a typical artificial neural network. In this study, MLP based on the back-propagation algorithm was used to train the prediction model. It consists of an input layer, an output layer and at least one hidden layer. Training includes two processes: signal forward propagation and error backward propagation. During forward propagation, input samples are transferred from the input layer to each hidden layer and output layer. Then, if the output value is not equal to the actual value, the backpropagation phase starts. By passing the output error back, the error is distributed to all the neural networks of the hidden layer, and the error of each layer of neurons is obtained as the basis for optimizing the weights of the neurons. Repeat the above process until the output error is acceptable or reaches the limit of the number of training iterations.

The nonlinear properties of the reed creep strain–time curve were considered to have initially identified the main structural parameters range of the MLP network, The range of MLP network structure hyper-parameters is shown in Table 3. In order to avoid unnecessarily increasing the complexity of the model, the number of the hidden layer is set to 1 or 2. The number of neurons is setting from 1 to 100. Subsequently, the MLP network prediction model of creep properties is constructed by optimizing the hyper-parameters of the model.

**Table 3:** The range of MLP network structure hyper-parameters

Hyper-Parameters	Range
Activation function	Logistic, Tanh, Relu
Number of hidden layers	1–2
Number of hidden layer neurons	1–10
Training method	L-BFGS, SGD, Adam

#### Support vector machine regression

As one of the most common methods in the machine learning field, support vector machine regression (SVR) has shown its unique advantages in solving the problems of small sample, nonlinear and high-dimensional pattern recognition. The basic idea of nonlinear SVR is to use nonlinear mapping $\phi$ to map data x to Hilbert feature space, then linear regression is carried out in this space. The kernel function $k\left( {x_{i} ,x_{j} } \right) = \phi \left( {x_{i} } \right) \cdot \phi \left( {x_{j} } \right)$ is used to realize the correspondence between linear regression in high-dimensional space and nonlinear regression in low-dimensional space. A three-order RBF kernel was considered in this study. The main hyper-parameter range of SVR was initially identified to include C and gamma (Table 4).

**Table 4:** The range of SVR hyper-parameters

Hyper-Parameters	Range
C	1–5000
gamma	[1 × 10^−5^, 0.1]

## Results

### Optimization results of hyper-parameters

The cross-validation method and genetic algorithm mentioned in Sect. 2.3.4 were used to optimize the hyper-parameters of the MLP and SVR machine learning prediction models. The results are shown as:

MLP: activation function = logistic, number of hidden layers = 2, number of hidden layer neurons = 8, training method = Adam;

SVR: C = 4298, gamma = 7.2 × 10^−4^.

**Fig.4:**
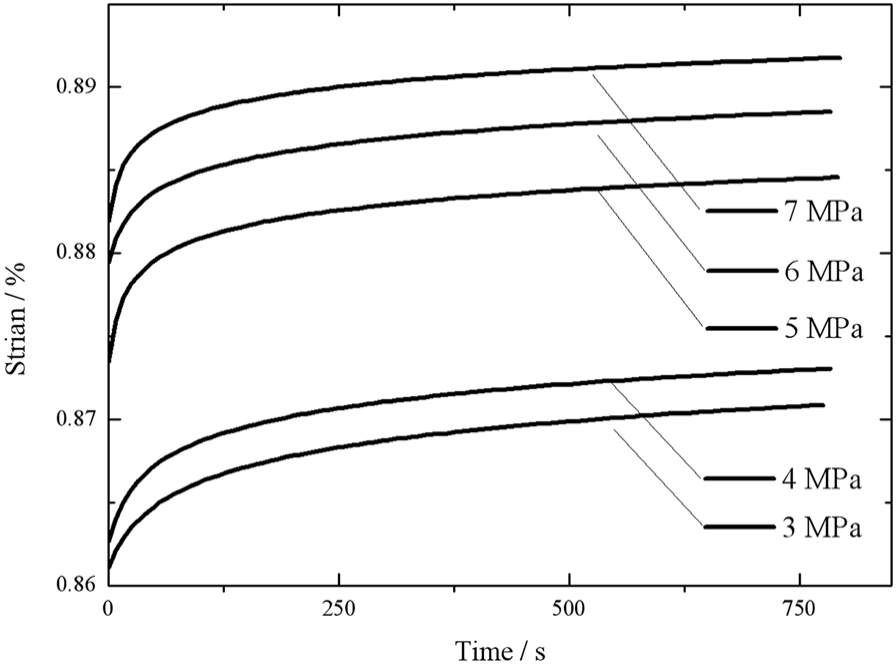
Reed bales vertical strain over time

Figure 6 shows the $\varepsilon$–*t* curves during the creep test process under various ${\sigma }_{0}$ s. As demonstrated in Fig. 4, the trends of $\varepsilon$ were consistent during all the tests. $\varepsilon$ changed at a high rate within the initial minute and subsequently at a decreasing rate. Overall, $\varepsilon$ increased nonlinearly at a high rate initially and subsequently at a low rate. During this process, as a result of the internal viscosity of the reeds, $\varepsilon$ continued to increase under constant $\sigma$. This process is the creep process of the reeds. The $\varepsilon$ values were calculated based on the test data using Eq. (2).

Additionally, as shown in Fig. 4, ${\sigma }_{0}$ affected the creep properties of the reeds. The higher ${\sigma }_{0}$ was, the higher the ultimate stable $\varepsilon$ (${\varepsilon }_{\infty }$) was.

**Fig. 5:**
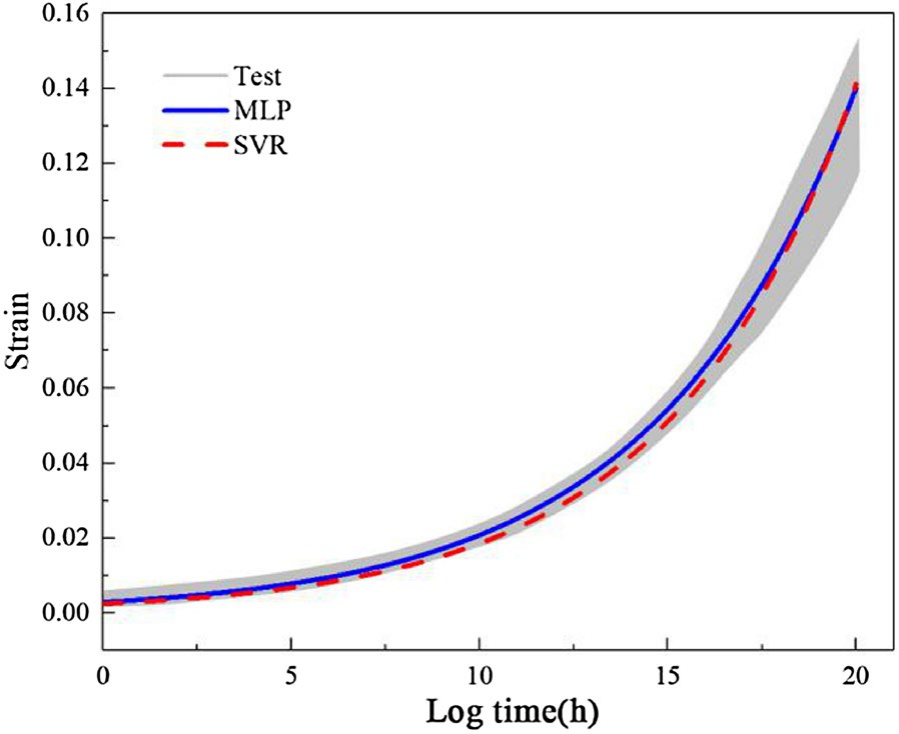
Comparison of MLP and SVR fitting and test results

In order to verify the fitting and prediction performance of the above model hyper-parameters, the creep curves under three loading forces are set as the training set, and the creep curve under another loading force is set as the prediction set. In addition, 10 training groups were also carried out. The result is shown in Fig. 5.

The blue and red lines in the Fig. are the creep curves predicted by MLP and SVR, respectively, and the shaded area refers to the predicted envelope range after 10 training sets. It can be seen that the two kinds of hyper parameter optimized machine learning models have good fitting and predictive performance on the creep curve of materials, and both can be used for creep performance prediction, but the MLP method is more accurate.

### Comparison of prediction performance under different applied forces

**Fig. 6:**
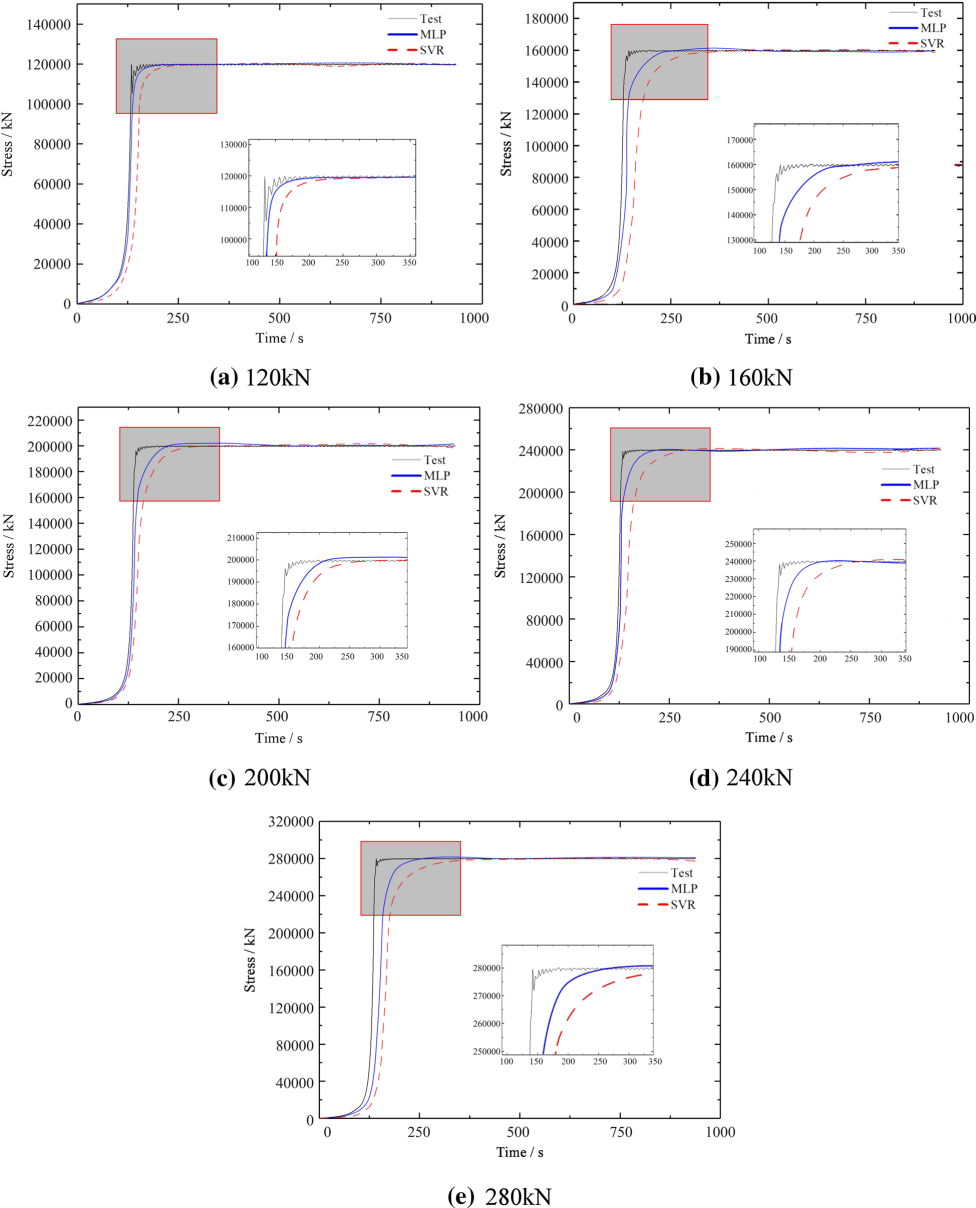
$\sigma$–*t* test and prediction curve under different forces. **a** 120kN. **b** 160kN. **c** 200kN. **d** 240kN. **e** 280kN

The medium pressure value fluctuates. Therefore, different applied forces will affect the compression efficiency of reeds, so it is necessary to further verify the prediction performance of MLP and SVR under different applied forces. During the reed compression process and the retention stage of the creep process, because the universal testing machine was unable to retain a completely constant $\sigma$, $\sigma$ fluctuated during the retention stage. Figure 6(a)–(e) shows the $\sigma$–*t* test and prediction curve under different forces. As demonstrated in Fig. 4, during the compression process, $\sigma$ changed exponentially with *t*. The compressive $\sigma$ fluctuated once reaching the preset maximum value. Within the initial minute, $\sigma$ fluctuated relatively significantly. As *t* increased, the fluctuations in $\sigma$ gradually weakened. The average $\sigma$ basically remained at *σ*_*0*_ ± 0.10 kN. In the first 60 s of the $\varepsilon$–*t* curves, the applied $\sigma$ fluctuated relatively significantly. During this process, a constant $\sigma$ was relatively difficult to maintain because of the relatively large reaction force from the compressed reeds as a result of the structural damage to and viscosity of the reeds. As *t* increased, the reaction force from the compressed reeds became relatively small, rendering it easier to maintain a constant $\sigma$. As a result, the applied $\sigma$ changed smoothly. Table 5 summarizes the changes in impulse during the five groups of tests. As the maximum compressive $\sigma$ increased, the corresponding impulse gradually increased.

**Table 5:** Impulse values under various $\sigma_{0}$ s

$\sigma_{0} ($ MPa)	3	4	5	6	7
Impulse (N × s)	9.72 × 10^7^	1.14 × 10^8^	1.62 × 10^8^	1.94 × 10^8^	2.27 × 10^8^

As shown in Table 6, under the action of five different forces, the fitting curve of the MLP model is better than the fitting curve of the support vector regression model. Under the force of 160kN and 280kN, the measured coefficient R^2^ of the fitted curve of the MLP model is 0.9115 and 0.9047, respectively, which means that there is a large deviation in the fitted curve. The fitting measurement coefficient R^2^ of the other three working conditions is greater than 0.997. At the same time, after comparing the MAE, RMSE, R and R^2^ of the two machine learning methods, MLP is superior. Therefore, under the simulation of the creep characteristics of reeds, the first task of the machine learning method is MLP.

**Table 6:** The generalization ability of two machine learning models

Applied Forces (kN)	Model	MAE	RMSE	R	R^2^
120	MLP	2.110 × 10^–3^	2.692 × 10^–3^	0.9992	0.9989
	SVR	1.417 × 10^–4^	1.612 × 10^–4^	0.9941	0.7730
160	MLP	9.878 × 10^–4^	1.354 × 10^–3^	0.9354	0.9115
	SVR	1.486 × 10^–4^	1.516 × 10^–4^	0.9939	0.6416
200	MLP	1.962 × 10^–3^	2.539 × 10^–3^	0.9989	0.9979
	SVR	1.294 × 10^–4^	1.374 × 10^–4^	0.9851	0.7058
240	MLP	2.158 × 10^–3^	2.675 × 10^–3^	0.9993	0.9985
	SVR	1.588 × 10^–4^	1.839 × 10^–4^	0.9892	0.8921
280	MLP	9.796 × 10^–4^	1.157 × 10^–3^	0.9252	0.9047
	SVR	4.144 × 10^–4^	4.378 × 10^–4^	0.9885	0.8759

### Reed material characteristics based on MLP model

The creep model parameters fitted to the creep test data obtained under various $\sigma_{0}$ s differ.${\text{ E}}_{{\text{m}}}$ reflects the elastic deformability of the reeds. The higher ${\text{E}}_{{\text{m}}}$ is, the lower the elastic deformability is, and the lower the compliance of the tissues inside the reed stalks is. In Table 5, ${\text{E}}_{{\text{m}}}$ ranges from 3.480 to 7.928 Pa, with an average of 5.715 Pa, an SD of 1.573 Pa and a CV of 27.52%. ${\text{E}}_{{\text{k}}}$ ranges from 500 to 1,166.667 Pa, with an average of 838.095 Pa, an SD of 235.895 Pa and a CV of 28.15%. ${\text{E}}_{{\text{m}}}$ is lower than ${\text{E}}_{{\text{k}}}$ in each case. This result suggests that of the internal structural models for the reeds, the elastic deformability of the elastic component (${\text{E}}_{{\text{m}}}$) is higher than that of the elastic component (${\text{E}}_{{\text{k}}}$) in the Kelvin model. The compliance of the elastic component in the Kelvin model is lower than that of the elastic model in the Maxwell model. The fitting coefficients of the MLP model are shown in Table 7.

**Table 7:** Fitting coefficients for the MLP models

Test No	$\sigma_{0}$(MPa)	Model coefficients				
		${\text{E}}_{{\text{m}}}$	${\text{E}}_{{\text{k}}}$	${\upeta }_{{\text{m}}}$	${\upeta }_{{\text{k}}}$	${\text{T}}_{{\text{k}}}$
1	3	3.480	500.000	0.685 $\times 10^{6}$	41,666.667	83.333
2	4	4.630	666.667	0.931 $\times 10^{6}$	47,619.048	71.429
3	5	5.682	1000.000	1.395 $\times 10^{6}$	66,666.667	66.667
4	6	6.857	857.143	1.546 $\times 10^{6}$	45,112.782	52.632
5	7	7.928	1166.667	2.034 $\times 10^{6}$	61,403.509	52.632
Average		5.715	838.095	1.318 $\times 10^{6}$	52,493.734	65.338
SD		1.573	235.895	0.474 $\times 10^{6}$	9754.204	11.710
CV		27.52%	28.15%	35.94%	18.58%	17.92%

${\upeta }_{{\text{m}}}$ reflects the deformation-resistant viscous resistance of the reeds. The higher ${\upeta }_{{\text{m}}}$ is, the higher the deformation-resistant viscous resistance is, and the poorer the fluidity of the internal structure is. In Table 5, ${\upeta }_{{\text{m}}}$ ranges from 0.685 to 2.034 Pa × s, with an average of 1.318 Pa × s, an SD of 0.474 Pa × s and a CV of 35.94%. ${\upeta }_{{\text{k}}}$ ranges from 41,666.667 to 61,403.509 Pa × s, with an average of 52,493.734 Pa × s, an SD of 9,754.204 Pa × s and a CV of 18.58%.

${\text{T}}_{{\text{k}}}$ reflects the time required for the components in the Kelvin model to reach strain equilibrium. In Table 5, ${\text{T}}_{{\text{k}}}$ ranges from 83.333 to 52.632 s, with an average of 65.338 s, an SD of 11.710 s and a CV of 17.92%. This result suggests that the average time for reaching creep equilibrium was 65.338 s.

As $\sigma_{0}$ increased, ${\text{E}}_{{\text{m}}}$, ${\text{E}}_{{\text{k}}}$, ${\upeta }_{{\text{m}}}$ and ${\upeta }_{{\text{k}}}$ all increased, whereas ${\text{T}}_{{\text{k}}}$ gradually decreased. This result suggests that the higher $\sigma_{0}$ was, the lower the elastic deformability was, the lower the compliance of the tissues inside the reeds was, the higher the deformation-resistant viscosity resistance was, the poorer the fluidity of the internal structure was, the higher the stability of the moulded reed bale was, and the shorter the time needed to reach creep equilibrium was.

The CV ranges from 17.92% to 35.94%, suggesting high dispersion. This result demonstrates relatively significant individual variation under various $\sigma_{0}$ s.

### Analysis of ${\mathbf{v}}_{{\mathbf{c}}}$ and ${\mathbf{J}}\left( {\mathbf{t}} \right)$

#### Analysis of v_c_

**Fig. 7:**
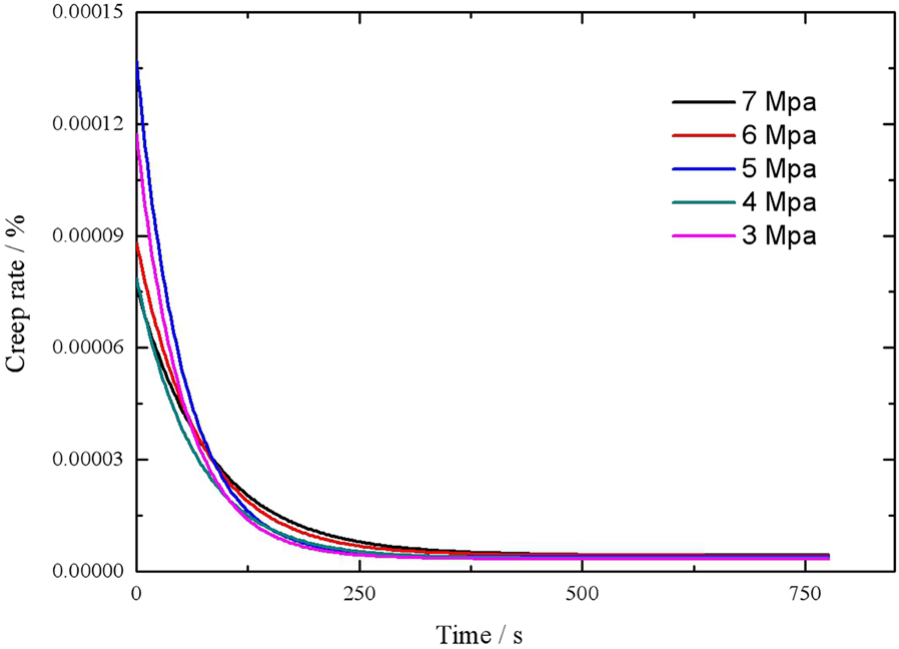
${\text{v}}_{c}$–*t* curves during the creep tests under various $\sigma_{0}$ s

The ${\text{v}}_{c}$ during the creep process of the reeds consists of a viscoelastic ${\text{v}}_{c}$ and a viscous ${\text{v}}_{c}$. Figure 7 shows the ${\text{v}}_{c}$–*t* curves during the creep process of the moulded reed bales.

As demonstrated in Fig. 7, the ${\text{v}}_{c}$ of the reeds exhibited similar trends under various $\sigma_{0}$ s. As *t* increased, ${\text{v}}_{c}$ was initially high, then deceased, and gradually tended to zero. Based on the changes in ${\text{v}}_{c}$, the creep process can be divided into three stages. During the first stage, ${\text{v}}_{c}$ was high. During this stage, the deformation is mainly composed of elastic deformation and viscous deformation, which was mainly a result of $\sigma_{0}$. From a microscopic perspective, the macromolecular chains of the elastic tissue structures (e.g., cellulose and lignin) in the reed stalks continued to extend, and the number of macromolecular bonds continued to increase. During this stage, the viscous creep resistance of the viscous tissue structures inside the reed stalks was relatively low. During the second stage, as *t* increased, ${\text{v}}_{c}$ changed at a low rate. During this stage, deformation consisted mainly of viscous deformation. This effect mainly occurred because the viscous resistance of the viscous tissue structures inside the reed stalks to continuous deformation gradually increased. As a result, continuous extension of macromolecular chains and increases in macromolecular bond angles inside the reed stalks were prevented. During the third and final stage, ${\text{v}}_{c}$ gradually decreased to a constant value. This stage was primarily characterized by irreversible plastic deformation. During this stage, the elastic dynamic force and the viscous resistance inside the reeds gradually reached equilibrium, and the tissues inside the moulded reed bale formed a new tissue structure. In the Kelvin creep model for the reeds, the elastic and viscous elements are mutually constrained. Additionally, when the elastic dynamic force increases, the viscous resistance decreases, and vice versa. Finally, the elastic dynamic force and the viscous resistance reached an equilibrium.

#### Analysis of J(t)

Calculated by formula (7) and MLP model, the creep compliance composition of the five sets of creep tests is shown in Table 8.

5 $$\varepsilon_{e} \left( t \right) = {\upsigma }_{0} J_{e} \left( t \right)$$

6 $$\varepsilon_{ve} \left( t \right) = {\upsigma }_{0} J_{ve} \left( t \right)$$

7 $$\varepsilon_{v} \left( t \right) = {\upsigma }_{0} J_{v} \left( t \right)$$

**Table 8:** Summarizes the compositions of ${\varvec{J}}\left( {\varvec{t}} \right)$ during the five groups of tests

${\upsigma }_{0}$	$J_{e} \left( t \right)$	$J_{ve} \left( t \right)$	$J_{v} \left( t \right)$
3	0.287	$0.002 \times 1 - {\text{e}}^{{ - 0.012{\text{t}}}}$	$1.46 \times 10^{ - 6}$
4	0.216	$0.0015 \times 1 - {\text{e}}^{{ - 0.014{\text{t}}}}$	$1.0745 \times 10^{ - 6} t$
5	0.175	$0.0014 \times 1 - {\text{e}}^{{ - 0.019{\text{t}}}}$	$7.762 \times 10^{ - 7} t$
6	0.147	$0.00083 \times 1 - {\text{e}}^{{ - 0.015{\text{t}}}}$	$5.973 \times 10^{ - 7} t$
7	0.126	$0.00086 \times 1 - {\text{e}}^{{ - 0.019{\text{t}}}}$	$4.916 \times 10^{ - 7} t$

Table 9 summarizes the various types of creep during the five sets of creep tests of the MLP model calculated according to Eqs. (6–7).

**Table 9:** Various types of ${\varvec{\varepsilon}}$ during the five groups of creep tests

${\upsigma }_{0}$	$\varepsilon_{e} \left( t \right)$	$\varepsilon_{ve} \left( t \right)$	$\varepsilon_{v} \left( t \right)$
3	0.861	$0.006 \times 1 - {\text{e}}^{{ - 0.012{\text{t}}}}$	$4.381 \times 10^{ - 6} t$
4	0.864	$0.006 \times 1 - {\text{e}}^{{ - 0.014{\text{t}}}}$	$4.298 \times 10^{ - 6} t$
5	0.875	$0.007 \times 1 - {\text{e}}^{{ - 0.019{\text{t}}}}$	$3.881 \times 10^{ - 6} t$
6	0.882	$0.005 \times 1 - {\text{e}}^{{ - 0.015{\text{t}}}}$	$3.584 \times 10^{ - 6} t$
7	0.882	$0.006 \times 1 - {\text{e}}^{{ - 0.019{\text{t}}}}$	$3.441 \times 10^{ - 6} t$

**Fig. 8:**
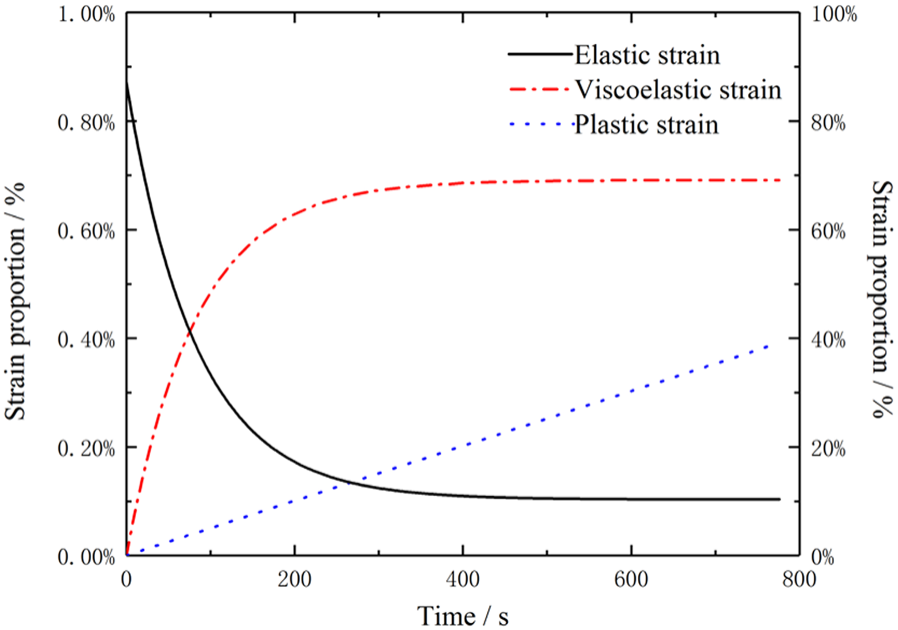
Proportion of each type of $\varepsilon$ in the total $\varepsilon$ during stress relaxation under a ${\sigma }_{0}$ of 3 MPa

The proportions of ${\varepsilon }_{e}\left(t\right)$, ${\varepsilon }_{ve}\left(t\right)$ and ${\varepsilon }_{v}\left(t\right)$ in the total $\varepsilon$ can be calculated using Eq. (7). Here, MLP is used as an example. Figure 8 shows the changes in the proportion of each type of $\varepsilon$ in the total $\varepsilon$ with *t* under a ${\sigma }_{0}$ of 3 MPa. The left axis shows the changes in the ${\varepsilon }_{e}\left(t\right)$ data. The right axis shows the changes in the ${\varepsilon }_{ve}\left(t\right)$ and ${\varepsilon }_{v}\left(t\right)$ data. The proportion of ${\varepsilon }_{e}\left(t\right)$ was the highest during the initial stage and gradually decreased to 99.19% with *t*. As *t* increased, the proportion of ${\varepsilon }_{ve}\left(t\right)$ increased rapidly initially and slowly subsequently. The proportion of ${\varepsilon }_{ve}\left(t\right)$ ultimately stabilized at 0.69%. The proportion of ${\varepsilon }_{v}\left(t\right)$ in the total $\varepsilon$ linearly increased with creep *t* and ultimately reached 0.39%. This result suggests that the degree of damage to the inherent structure of the reeds gradually increased with *t*.

## Discussions

### Motivations

We studied the creep behaviour of reed bales under different holding forces. The test curves are fitted by Machine Learning Prediction Algorithms and Support Vector Machine Regression, and the constitutive equations and constitutive coefficients of the reed creep process are obtained. In addition, a four-element creep model of Reed was established using the Machine Learning Prediction Algorithms model. The results show that the creep behaviour of a reed bale was positively correlated with the initial maximum compressive stress (*σ*_*0*_). The established Burgers four-element model was capable of simulating the creep process of reed bales. The test curves coincided well with the model-simulated curves. Reed bales were found to exhibit viscoelasticity. During the creep process, the elastic dynamic force and the viscous resistance were mutually constrained. The ε of reeds was composed of elastic, viscoelastic and plastic *ε*. The creep process could be divided into three stages. During the first stage, σ_0_ was constant. During the second stage, *ε* rapidly increased within one minute. During the last stage, ε slowly increased with t, and the displacement ultimately reached a stable value.

### Limitations

The creep characteristics of the reed are closely related to the compression time, strain value, stress and delay time, showing strong nonlinear characteristics. The MLP model is better than the SVR model in predicting MAE, RMSE, R and R^2^ under different forces, and the R^2^ of MLP is all greater than 0.9. The method described in this study is an application case of machine learning technology in the study of material properties, which can provide new research ideas for the accelerated characterization of material mechanical properties.

Although a new idea for predicting the compression creep performance of reeds is proposed, the author believes that the current research still has the following limitations:

- Few experimental working conditions are considered, and the total data set is relatively small. With larger data sets, the accuracy and reliability of machine learning models such as MLP and SVR will be improved;
- Only four more important creep-related variables are considered, including compression time, strain value, stress and delay time. However, the creep characteristics of reeds are more complicated. Therefore, follow-up research should involve more variables to further optimize the MLP prediction model.

## Future work

In order to further improve the fitting accuracy of the training set, the hyper parameter optimization method of the machine learning model combining genetic algorithm and k-fold cross-validation will be studied:

- Introduce more variables, such as pattern size and material thermal properties, to optimize the prediction model more comprehensively;
- Enhance the inherent laws of patterns under different working conditions, further strengthen nonlinear characteristics, and improve prediction accuracy.

## Conclusions

This study uses a series of machine learning methods (based on MLP and SVR) to predict the compressive creep deformation of reeds. Considering variables such as compression time, strain value, stress and delay time, a compression creep test was carried out on the reed samples. Using the established MLP model to analyze the parameters of creep rate and creep compliance, it verified the model's fitting accuracy in the training set and its predictive ability under new conditions. According to the results, the following conclusions can be drawn: There is an irreversible plastic strain in the creep process of the reed block, and the creep process is a process in which elastic dynamics and viscous resistance are restrained by each other. In the experiments of different loading forces, the creep process trend of reed is the same. The strain change rate is fast in the first minute, and then it becomes slow, showing a non-linear growth trend of fast first and then slow overall. Different loading stresses have an impact on the creep of reeds. The greater the loading force, the greater the final stable strain value. According to the creep characteristic parameters, the Burgers four-element model established by the virtual prototype software can simulate the creep process of the reed block. When the MLP model simulates the strain of the reed under different pressures, the R^2^ is greater than 0.9, and the simulation curve has the same trend as the experimental curve, and the overlap effect is good. The stress of reed creep is mainly composed of elastic strain, viscoelastic strain and plastic strain. Among them, the elastic strain accounts for the largest proportion, and it decreases with the increase of time. The viscoelastic strain first increases and then becomes stable with the increase of time. The strain increases linearly with time, and the damage to the structure of the reed gradually increases. The method described in this study is an application case of machine learning technology in the study of material characteristics, which can provide new research ideas for the accelerated characterization of material mechanical properties.

## Data Availability

The datasets used and analyzed during the current study are available from the corresponding author on reasonable request.
